# Integrating TB and non-communicable diseases services: Pilot experience of screening for diabetes and hypertension in patients with Tuberculosis in Luanda, Angola

**DOI:** 10.1371/journal.pone.0218052

**Published:** 2019-07-05

**Authors:** Giulia Segafredo, Anil Kapur, Claudia Robbiati, Nsuka Joseph, Joseth Rita de Sousa, Giovanni Putoto, Fabio Manenti, Andrea Atzori, Ugo Fedeli

**Affiliations:** 1 Planning and Operational Research Unit, Doctors with Africa CUAMM, Padova, Italy; 2 World Diabetes Foundation, Bagsvaerd, Denmark; 3 Program Department, Doctors with Africa CUAMM Angola, Luanda, Angola; 4 DAT TB Dispensary, Luanda, Angola; 5 Non Communicable Diseases Section, Ministry of Health, Luanda, Angola; 6 Program Department, Doctors with Africa CUAMM, Padova, Italy; 7 International Relations Department, Doctors with Africa CUAMM, Padova, Italy; 8 Epidemiological Department (SER), Azienda Zero, Padova, Italy; Agha Khan University, UNITED REPUBLIC OF TANZANIA

## Abstract

**Background:**

In the face of the rising burden of non-communicable diseases like diabetes mellitus (DM) and hypertension in sub-Saharan Africa, where infectious diseases like Tuberculosis (TB) are still endemic, the double burden of communicable and non-communicable diseases appears to be increasing rapidly. However, the size of the problem and what is the proper health system approach to deal with the double burden is still unclear. The aim of this project was to estimate the double burden of DM hypertension and TB and to pilot the integration of the screening for DM and hypertension in the TB national programs in six TB centers in Luanda, Angola.

**Methods:**

All newly diagnosed pulmonary TB (PTB) patients accessing six directly observed treatment (DOT) centers in Luanda were screened for diabetes and hypertension. TB diagnosis was made clinically and/or with sputum microscopy DM diagnosis was made through estimation of either fasting plasma glucose (FPG) (considered positive if ≥ 7∙0mmol/l) or random plasma glucose (considered positive if ≥ 11∙1mmol/l). Uncontrolled hypertension was defined as systolic blood pressure (SBP) of ≥ 140 mm of Hg and/or diastolic blood pressure (DBP) of ≥ 90 mm of Hg, irrespective of use of antihypertensive drug.

**Results:**

Between January 2015 and December 2016, a total of 7,205 newly diagnosed patients with PTB were included in the analysis; 3,598 (49∙9%) were males and 3,607 females. Among 7,205 PTB patients enrolled, blood pressure was measured in 6,954 and 1,352 (19∙4%) were found to have uncontrolled hypertension, more frequently in females (23%) compared to males (16%). In multivariate logistic regression analysis uncontrolled hypertension was associated with increasing age and BMI and ethnic group. The crude prevalence of DM among TB patients was close to 6%, slightly higher in males (6∙3%) compared to females (5∙7%). Age adjusted prevalence was 8%. Impaired fasting glucose (>6∙1 to <7∙0 mmol/L) was detected in 414 patients (7%). In multivariate logistic regression analysis DM prevalence was higher in males and increased with increasing age and BMI.

**Interpretation:**

TB patients have a considerable hypertension and diabetes co-morbidity. It is possible to screen for these conditions within the DOTs centres. Integration of health services for both communicable and non-communicable diseases is desirable and recommended.

## Introduction

Despite substantial progress in the last two decades, Tuberculosis (TB) remains a considerable global public health concern, particularly among the poor and vulnerable populations [1]. However, even if low and middle-income countries still struggle to gain control over communicable diseases, they are being confronted with a new health-challenge. Non-communicable diseases (NCDs) are now the leading global cause of death and are responsible for 70% of deaths worldwide and approximately 80% of all NCD deaths in 2008 occurred in low and middle-income countries also prematurely. [2]

Rapid demographic, sociocultural, nutrition and economic transitions are driving an increase in the risk and prevalence of NCDs, such as diabetes, cardiovascular diseases and cancer, especially in sub-Saharan Africa. [3,4]

The 2017 Global Burden of Disease, showed a 2-fold increase in terms of disability-adjusted life year (DALYs) and deaths attributable to diabetes between 1990 and 2017 in sub-Saharan Africa [5]. The impact of this transitions and their health consequences, therefore, are going to be massive and health-systems, still very fragile, will need to find effective and sustainable approaches to address the multi-faceted challenge of infectious diseases while also addressing NCDs. [6] In order to better understand how to address this double burden, it is important to improve the understanding of how communicable and non-communicable diseases are linked. Questions regarding the burden of the co-morbidity, the increased risk that the co-morbidity imposes, what sustainable health-system approaches can be taken to address both communicable and non-communicable diseases, especially in fragile countries, are not yet fully answered. The link between tuberculosis and diabetes has been widely described. The evidence showing the association between the two diseases is now robust and WHO refers to the interaction as an ‘intersecting epidemic’. [7] Diabetes increases the risk of active tuberculosis, of progression from latent to active infection and of TB transmission. In addition to this, DM patients have a poorer response to TB treatment, resulting in a higher risk of treatment failure and, as a consequence, a higher risk of worse TB outcome. [1,7–9] On the other hand, being a systemic infection, TB can worsen glycemic control and make the clinical management of DM more complicated. [10,11] However, diabetes is not the only disease, among NCDs, with an increasing prevalence in sub saharan Africa. Cardiovascular diseases (CVD) are expected to be the biggest cause of death for most developing countries by 2020, similar to the current epidemiology of CVD in developed nations. [12,13] The increase in prevalence of traditional risk factors such as obesity, kidney diseases and hypertension explains much of this increase, but studies indicate that the burden of infectious diseases may also contribute to the development of CVD. [13,14, 15] The relationship between hypertension and TB is less clear than the one between TB and DM. [16]. However, it has been suggested that the triggering of immunological response due to a systemic infection, can cause an impairment of the endothelial function and increase the risk of CVD and, possibly, hypertension. [17,18] On the other hand, hypertension may have some effect on the immune system. [19] Irrespective of what is the actual pathological pathway that links TB and NCDs, it clearly appears that in the coming years, Sub-Saharan Africa will face the challenge of dealing with high burden of infectious diseases while also needing to address the increasing burden of NCDs. [6] Bidirectional screening and integrated management can help to improve early diagnosis and health outcomes for both conditions [1,7] but there is inadequate evidence, so far, on the feasibility and effectiveness of this approach.

Doctors with Africa CUAMM, the first Italian non-governmental organization, carried out this pilot project, aimed at defining the burden of co-morbidity and to explore the feasibility of integrating screening for diabetes and uncontrolled hypertension in newly diagnosed TB patients in an urban population of Luanda the capital city of Angola, receiving treatment within the Angolan National Tuberculosis program.

## Materials and methods

### Settings

The project was carried out in Luanda, the capital of Angola. According to the last census, about 3 million inhabitants live in the capital, although unofficial sources estimate that at least one third of the country population (30 millions) lives in the capital. The National TB Prevention and Control Program (NTP) relies on Directly Observed Therapy, Short-Course (DOTS) centers for the detection and follow up of TB patients. Six DOTS centers were purposively included in the project, namely: Hospital Divina Providência, Municipality of Kilamba Kiaxi; Hospital do Sanatório, Municipality of Kilamba Kiaxi; Centro de saúde de Cacuaco, Municipality of Cacuaco; Centro de Saúde do Cariango, Municipality of Cazenga; Dat-Dispensário Anti Tuberculosis Municipality of Luanda; Centro de Saúde da Boa Nova, Municipality of Viana. By virtue of their location in the city, the 6 DOTS centers provide a representative sample of the urban population of Angola.

### Patients and procedures

All newly diagnosed TB patients aged ≥ 15 years who attended one of the six DOT centers from January 2015 to December 2016 were offered the opportunity to be screened for DM and hypertension and sensitization about TB comorbidities. Sample size was not pre-determined as the screening was offered to all patients attending the selected clinics. Patients unwilling to participate or unable to give informed consent were excluded.

Once that the patient accepted to participate, a structured questionnaire addressing socio-demographic and clinical information was administered by the study personnel (nurses and LAB technician). (S1 and S2 Files) After the questionnaire was completed patients underwent clinical examination.

Capillary blood glucose was determined using STATSTRIP XPRESS GLU/KET (Nova Biomedica) blood glucose meters which are calibrated to provide plasma equivalent results.

Based on the time from the last food intake, capillary blood glucose was considered fasting glucose, FBG, if >8 hours had elapsed since last meal or capillary random glucose, RBG, if the last meal was <8 hours at the time of blood collection.

A patient was considered as having DM if at least one of these criteria was satisfied: fasting plasma glucose ≥126 mg/dL; random plasma glucose ≥200 mg/dL; previous diagnosis of DM. We considered impaired glucose tolerance if FBS was ≥110 mg/dL, but <126 mg/dL. Blood pressure was measured using an automatic digital blood pressure monitor: PIC Classic Check (Artsana S.p.a). Uncontrolled blood pressure was defined as diastolic blood pressure ≥ 90 mm of Hg and/or a systolic blood pressure ≥ 140 mm of Hg, irrespective of use of any antihypertensive drug. Weight and height were measured with Eye-Level Mechanical Physician Scales Cardinal DETECTO 2391 (Cardinal Scale Manufacturing Co.). BMI was calculated according to the WHO international classification and patients were considered underweight if BMI was <18∙5 kg/m2, within the normal range if BMI was 18∙5–24∙9 kg/m2, overweight if BMI 25–29∙9 kg/m2, and obese if BMI ≥30 kg/m2. [19]

After clinical examination the patient received proper and exhaustive information on risk factors for DM-HTN and healthy lifestyle by community health workers at DOTS centres.

### Statistical analysis

Associations between categorical variables were assessed by means of the Chi-square test. Demographic and clinical factors significantly associated with presence of diabetes mellitus or to uncontrolled hypertension were selected by means of stepwise logistic regression models constrained to keep gender and age as determinants; the final models provided the associated Odds Ratios with 95% Confidence Intervals.

### Ethical approval

According the National regulation, the protocol was approved by the ethical committee of the National Directorate of Public Health. All patients provided a written informed consent before inclusion in the study.

### Role of funding source

The funding source did not participate to study design, data collection and analysis or the interpretation of data.

The corresponding author had full access to all the data in the study and had final responsibility for the decision to submit for publication.

## Results

Between January 2015 and December 2016, a total of 7,205 newly diagnosed TB patients were included in the analysis, of which, 3,598 (49,9%) were males and 3,607 females.

759 patients were enrolled at the Hospital Divina Providência, 540 in the Hospital do Sanatório, 815 in the Centro de saúde de Cacuaco, 1,714 in the Centro de Saúde do Cariango, 2,103 in the Dat-Dispensário Anti Tuberculosis and 1,274 Centro de Saúde da Boa Nova.

62% patients were smear positive, and 48% of reported at least four TB-related symptoms (cough, hemoptysis, fever, asthenia, dyspnea, weight loss, night sweat, chest pain).

30% of the patients were in the 25–34 year-age-category and 54% belonged to the the Kimbundu etnic group. 61% of the study population had at least a secondary level of education and illiteracy was more frequent in females.

Smoking and alcohol consumption were more frequent among males than females. Table 1 provides the characteristics of the study subjects.

**Table 1:** Characteristics of the study population overall and segregated by gender. n is the absolute number (%) indicate the proportion in the general population or among male/female population.

	n (%)	Males, n (%)	Females, n (%)
**Age, yrs (n = 7,205)**			
15–24	2,001 (28)	1,002 (28)	999 (28)
25–34	2,143 (30)	1,125 (31)	1,018 (28)
35–44	1,504 (21)	747 (21)	757 (21)
45–54	842 (12)	394 (11)	448 (12)
≥55	715 (10)	330 (9)	385 (11)
**Ethnicity (n = 7,081)**			
Kimbundu	3,809 (54)	1,822 (52)	1,987 (56)
Umbundo	1,402 (20)	722 (20)	680 (19)
Bacongo	1,071 (20)	573 (16)	498 (14)
Other	799 (11)	416 (12)	383 (11)
**Education (n = 6,596)**			
Illitterate	658 (10)	143 (4)	515 (16)
Primary	1,872 (28)	799 (24)	1,073 (32)
Secondary, I cicle	2,187 (33)	1,256 (38)	931 (28)
Diploma and above	1,879 (28)	1,091 (33)	788 (24)
**Smoking status (n = 7,132)**			
Never smoker	6,261 (88)	2,828 (79)	3,433 (96)
Ever smoker	871 (12)	730 (21)	141 (4)
**Alcohol (n = 7,150)**			
Non-drinker	4,961 (69)	2,044 (57)	2,917 (81)
Drinker	2,189 (31)	1,523 (43)	666 (19)
**Clinical TB score (n = 7,135)**			
<4	3,718 (52)	1,770 (50)	1,948 (55)
≥4	3,417 (48)	1,794 (50)	1,623 (45)
**Smear positive (n = 6,055)**			
No	2,309 (38)	1,019 (33)	1,290 (44)
Yes	3,746 (62)	2,113 (67)	1,633 (56)
**Prev. diagnosis of hypertension (n = 7,205)**			
No	5,920 (82)	3,170 (88)	2,750 (76)
Yes	1,285 (18)	428 (12)	857 (24)
**Prev. diagnosis of diabetes (n = 7,205)**			
No	7,144 (99)	3,565 (99)	3,579 (99)
Yes	61 (1)	33 (1)	28 (1)
**BMI, kg/m**^**2**^ **(n = 6,866)**			
<16	785 (11)	454 (13)	331 (10)
16–18.4	2,029 (29)	1,194 (35)	835 (24)
18.5–24.9	3,166 (46)	1,586 (46)	1,580 (46)
25–29.9	566 (8)	162 (5)	404 (12)
≥30	340 (5)	60 (2)	280 (8)
**High blood pressure (n = 6,954)**			
No	5,602 (81)	2,926 (84)	2,676 (77)
Yes	1,352 (19)	569 (16)	783 (23)

Previous diagnosis of hypertension and DM was reported by 18% and 1% of the study subjects respectively. As regards BMI class distribution, 40% of the patients were underweight (more men than women) while 13% were overweight or obese (more women than men).

Among 7,205 TB patients enrolled, 6,954 had valid documented blood pressure record. Of these 1,352 (19∙4%) had uncontrolled blood pressure, more women (23%) compared to men (16%). In multivariate logistic regression analysis hypertension was associated with age, BMI and ethnic group. (Table 2)

**Table 2:** Factors associated with the probability of having uncontrolled blood pressure. Odds Ratios estimated by logistic regression analysis.

	Odds Ratio	[95% Conf.Interval]
Age 15–25	1	-
Age 25–35	**1**∙**66**	[1∙31–2∙11]*
Age 35–45	**2**∙**82**	[2∙23–3∙57]*
Age 45–55	**6**∙**30**	[4∙92–8∙07]*
Age ≥55	**10**∙**78**	[8∙38–13∙87]*
Male gender	1∙00	-
Female Gender	1∙07	[0∙93–1∙23]
BMI (kg/m2)	**1**∙**12**	[1∙10–1∙13]*
Kimbundu	1	-
Umbundo	**0**∙**74**	[0∙61–0∙89]*
Bacongo	**0**∙**71**	[0∙58–0∙87]*
Other	1∙00	[0∙8–1∙25]*

*p value < 0.05.

Four-hundred-thirty-one TB patients satisfied at least one of the criteria for DM; of these 14% were known cases of DM and 86% were newly diagnosed on testing. The crude prevalence rate for DM was 6%, slightly higher in males (6∙3%) than females (5∙7%). When DM prevalence was age-standardized (WHO 2011 criteria), it reached 8%.

368 (85%) patients were diagnosed based on raised fasting plasma glucose, 23 patients resulted to have random blood sugar ≥200 and only 61 patients with DM were previously diagnosed for the disease (Table 3).

**Table 3:** Prevalence of previous diagnosis of DM, FBS, RBS.

Study variable	N	%
**Previous diagnosis of diabetes (n = 7,205)**		
No	7,144	99%
Yes	61	1%
**Fasting blood sugar (n = 5,890)**		
<110	5108	86∙5%
110–125	414	7%
≥126	368	6∙5%
**Random blood sugar (n = 1,174)**		
<200	1151	98%
≥200	23	2%

Impaired fasting glucose (IFG) was detected in 414 patients (7%). Thus almost 12% of the study population had dysglycaemia.

The prevalence of diabetes increased with age and lower literacy. Minor variations were found based on ethnicity, with Kimbundu and Makongo ethnic groups displaying a higher prevalence. A slightly higher prevalence of DM was seen among TB patients who smoked.

The prevalence of DM was two-times higher among subjects with uncontrolled hypertension, and was significantly higher in overweight or obese subjects (Table 4).

**Table 4:** Prevalence of DM by main study variables.

	N	% diabetes
**Gender**		
Male	3,598	6∙3%
Female	3,607	5∙7%
**Age, yrs**		
15–24	2,001	**2**∙4%*
25–34	2,143	**3**∙5%*
35–44	1,504	**7**∙4%*
45–54	842	**12**∙0%*
≥55	715	**13**∙3%*
**Ethnicity**		
Kimbundu	3,809	6∙3%
Umbundo	1,402	5∙2%
Bacongo	1,071	6∙5%
Other	799	5∙5%
**Education**		
Illitterate	658	**9**∙3%*
Primary	1,872	**7**∙7%*
Secondary, I cicle	2,187	**5**∙0%*
Diploma and above	1,879	**4**∙1%*
**Smoking status**		
Never smoker	6,261	5∙9%
Ever smoker	871	6∙4%
**Alcohol**		
Non-drinker	4,961	6∙0%
Drinker	2,189	6∙0%
**BMI, kg/m**^**2**^		
<16	785	**5**∙4%*
16–18.4	2,029	**4**∙4%*
18.5–24.9	3,166	**5**∙5%*
25–29.9	566	**9**∙5%*
≥30	340	**12**∙1%*
**High blood pressure**		
No	5,602	**4**∙9%*
Yes	1,352	**10**∙1%*

*p value < 0.05

In multivariate logistic regression analysis DM prevalence was higher in males than females and increased with increasing age and BMI (Table 5).

**Table 5:** Factors associated with the probability of having DM. Odds Ratios estimated by logistic regression analysis.

	Odds Ratio	[95% Conf.Interval]
Age 15–24	1	-
Age 25–34	**1**∙**51**	[1∙03–2∙20]*
Age 35–44	**3**∙**14**	[2∙19–4∙52]*
Age 45–54	**5**∙**19**	[3∙56–7∙56]*
Age ≥55	**5**∙**70**	[3∙89–8∙35]*
Male gender	1∙00	-
Female Gender	**0**∙**75**	[0∙60–0∙93]*
BMI (Kg/m2)	**1**∙**03**	[1∙01–1∙05]*

*p value < 0.05

## Discussion

The aim of the project was to explore the feasibility of integrating screening activities for uncontrolled hypertension and diabetes mellitus within routine TB activities rolled out by the NTP and to better understand what is the burden of the two conditions among newly diagnosed TB patients in an urban population of Luanda, the capital of Angola.

The crude prevalence of DM among TB patients observed in the selected DOTS centres in Luanda was 6∙3% for males and 5∙7% for females, and the age-standardized prevalence was 8%. The International Diabetes Federation [20] estimates a prevalence of 3∙2% in the general Angolan population, however further national data report figures ranging from 2∙8%-3∙3% [21] to 5∙9% [22,23]. The difference in terms of population tested and the wide range of prevalence data, make the understanding of the actual burden very difficult. Despite this uncertainty, our findings suggest that TB population could have a higher risk of DM compared to that of the general (Non-TB) population as reported from other developing countries both in Africa [24,25] and other parts of the world. [26–28] Only 1% of the newly diagnosed TB population had previously known DM, underlining the importance and utility of integrating screening for diabetes in patients with TB. Patients with TB and DM tended to be older and had a higher BMI, which is in line with what is already known.

In addition 414 patients had impaired fasting glucose, which is associated with impaired suppression of hepatic glucose output and impaired insulin secretion and is considered a precursor for diabetes (a pre-diabetes stage), suggesting a high risk for future DM in this population.

Almost one in five (19∙4%) of the newly diagnosed TB patients also had uncontrolled hypertension. The prevalence rose to one in three (33%) in TB patients with co-morbid DM. The prevalence of hypertension among newly diagnosed TB patients in our study is similar to that reported by Pires et al. in a community-based survey also in an urban setting in Dande in northern Angola. [29] They reported a 23% prevalence of hypertension and, as in our data, HTN seemed to be significantly associated to age and BMI and inversely associated with the level of education. Other studies have reported a much higher rate of hypertension in the general population in Angola. Paquissi et al. reported hypertension prevalence of 38∙5% in patients attending the outpatient clinic of the general hospital of Huambo [30]. In this study hypertension was significantly associated with age and female gender. Evaristo-Neto carried out a cross-sectional study in a rural community in Bengo found 38∙7% prevalence of hypertension in this community [21]. Lastly, Capingana et al. detected a hypertension prevalence of 45% of among public workers in Luanda. [23] All these data confirm the generally high burden of hypertension in the general population of Angola. Our data does not suggest any linkage between TB and higher prevalence of hypertension per se other than through the linkage of DM and hypertension.

The project underwent through several implementation challenges that should be taken into consideration in case a scale up is planned. To our knowledge, this was the first experience of integration of the two services within the country. Therefore, no national guidelines or protocols are available for the integrated diagnosis and management of the two diseases. This could be overcome through the creation of a functional TB/DM working group at national level to develop clear strategies and structure a common training of TB and DM healthcare workers. Integration of the two services could be further exploited to improve the follow up of DM patients which resulted very difficult both for the lack of human resources dedicated to the recall of the patients but also for the lack of free services and for the national stock out of medication both for TB and DM.

Finally, data collection system and data quality should be strengthened, the creation of a common framework of key performance indicators could be helpful to achieve these objectives.

Our study had a few limitations. Despite the substantial size of the study sample, we cannot consider our data fully representative of the general population of Angola. DOTs centres involved in the project are located in urban areas, therefore rural population was excluded. Only TB patients referred to DOTs center were invited to the screening, so TB patients not referred to health services are not represented. Our population only included TB patients, we did not study a control population without TB so our data does not allow direct comparison of DM and hypertension prevalence between TB and non-TB patients. We used glucometers to measure blood glucose and this certainly is not the most appropriate way to diagnose DM and blood glucose measurement was only done once. This approach was adopted because this is perhaps the only feasible way in low-resource settings and has been used in other studies in the developing world. However, to our knowledge, this is the first analysis of the TB–NCD co morbidity ever made in Angola.

Although further evidence needs to be generated, our study shows the considerable high burden of comorbidity of TB, DM and hypertension in an urban setting in Angola. This double-burden (infectious and non-communicable diseases) represents a huge public health challenge which requires more research to better understand the association between TB and NCDs and to develop a model of care that includes primary prevention and health education activities and is able to provide integrated health-services for both communicable and non communicable health conditions. Our pilot project demonstrates that integration of TB program and NCDs activities for the detection of DM and hypertension is feasible and confirms the need of further strengthening the implementation of policies, guidelines and monitoring and ensuring availability of medicines, basic technologies and procedures in the public health sector to address the double burden of TB and DM as advocated in the Bali Declaration. [31]

## Supporting information

S1 File Survey questionnaire English. (DOC)

S2 File Survey questionnaire Portuguese. (DOC)

S3 File Anonymized data set. (XLSX)
