# Supplementary material for: Integrating TB and non-communicable diseases services: Pilot experience of screening for diabetes and hypertension in patients with Tuberculosis in Luanda, Angola
Source: PLoS One. 2019 Jul 5;14(7):e0218052. doi: 10.1371/journal.pone.0218052 (PMC6611589; doi:10.1371/journal.pone.0218052)
Supplement: S1 File — (DOC) [file pone.0218052.s001.doc]

**
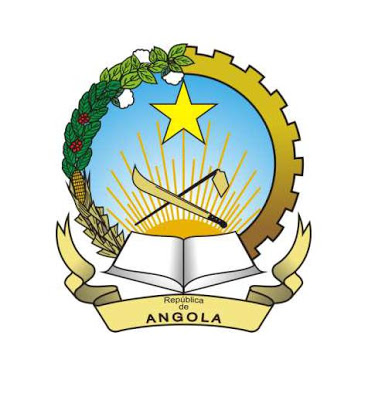
**

**República de Angola**

***National Tuberculosis Program***

**Poject:**

**IMPROVE THE DIAGNOSIS OF DIABETES AND HYPERTENSION IN PATIENTS WITH TUBERCOLOSIS**

Date of registration: ………… /…………… /…………………

Name of the health center: .......................................

Municipality: .............................................................

District: ......................................................................

**Personal data**

Patient identification number: ………………………………………....

Name: ………………………………………………………………………........................... Sex: F M

Date of birth: ……… /……………. / …………….. Age: ……………………..

Address: …………………………………………………………………………………………………………………………………………….

nº ……………. Block: ………………………………………. Zone: ……………………

Municipality: …………………………………………………………..

City: …………………………………………...... Telephone numer: ………………………………………...............

Name of the caregiver: ……………………………………………………………………………………………………………………

Type of relationship: ………………………………………. Telephone number: …………………………………………………..

**Patient informed consent**

Do you consent to participate to the study? Yes No

Have you have already been tested for glycamenia? Yes No

Are you a diabetec patient? Yes No

1/4

**Socio- demographic information**

Civil status: Single Married Divorced

Widower Living with a partner

Type of employment :

Employee Unemployed Housewive

Estudante Other

Ethnicity: Kimbundu Umbundo Bacongo Other

Religion : Christian Muslim Hindu Other

Education: Illiterate Primary school

Secondary school

University

Number of children: 0 1 2 ≥3

Income (monthly income in Kwanzas): …………………………………………

Number of people living in the household: 1-5 6-8 ≥9

Smoke? No Yes, ≤ 1 year Yes, 1-5 years Yes, ≥10 years

Do you dring alcoholic drinks? No Sometimes Everyday

**Clinical information on TB/HIV**

Have you ever had TB treatment? Yes No

Do you have any of these symptoms? Cough Hemoptysis Fever

Asthenia Dispnoea Weight loss

Night sweats Thoracalgia

Have you ever tested for HIV? Yes No

Are you on ARV? Yes No

If yes, what type of ARV are you taking…………………………………………………………………………………………

2/4

**Clinical information on diabetes**

Do you know what diabetes is? Yes No

Are you on treatment? Yes No

Type of treatment ………………………………………………………………………………………………………………………………

Since when ……………………………………………………………………………………………………………………………….

Do you have any diabetes relative Yes No

Do you have any of these symptoms? Polyphagia Polyuria Polydipsia Weakness

Did you eat today? Yes No

At what time? ……………………………………………………………………………………………………………………………………....

**Clinical information on blood pressure**

Do you suffer of high blood pressure? Yes No

Are you under treatment? Yes No

Does anyone suffer of hypertension in your familty? Yes No

**Additional clinical information**

Do you have other diseases? Yes No

What? …………………………………………………………………………………………………………………………………………………...

……………………………………………………………………………………………………………………………………………………………..

3/4

**Diagnostic information**

Patient identification number in the Laboratory TB register: ……………………………..

Smear Microscopy : BK+ BK− BK N/R

Microscopy: Negative 1-9 + ++ +++

HIV Test: HIV + HIV – HIV N/R

Blood pressure: Sistolic: ………... mm/Hg Diastolic: ………… mm/hg Heart frequency: ………….P.

**[Threshold : 140 mm/Hg Threshold: 90 mm/hg]**

Anthropometric: Weight: …….......... kg Height: ….........…. cm

Abdominal circunference: ............... cm BMI (weight/height²): ...............

Diabetes: Time of glycaemia test:

Result: …….......... mg/dl **[N.V. ˂110 mg/dl]**

**Follow up**

**If glycaemia ˃ 126 mg/dl:**

Refer the patient to the Health Facility named ……………………………………………….....................................

**If the blood pressure is ˃ 140/90 mm/Hg**

Refer the patient to the Health Facility named..………………………………………………………………...…………….

**Observations**

…………………………………………………………………………………………………………………………………………………………………………………………………………………………………………………………………………………………………………………………

**NB: If the patient has not been fasting for 8 hours, perform the test anyway and inform him that the test will be repeated on the day of the smear result and the patient must arrive at the center having fasted for at least 8 hours.**

**Name of the responsible**  **Signature**

……………………………………………………………… …………………………………………………………

4/4
