# Supplementary material for: Integrating TB and non-communicable diseases services: Pilot experience of screening for diabetes and hypertension in patients with Tuberculosis in Luanda, Angola
Source: PLoS One. 2019 Jul 5;14(7):e0218052. doi: 10.1371/journal.pone.0218052 (PMC6611589; doi:10.1371/journal.pone.0218052)
Supplement: S2 File — (DOC) [file pone.0218052.s002.doc]

**
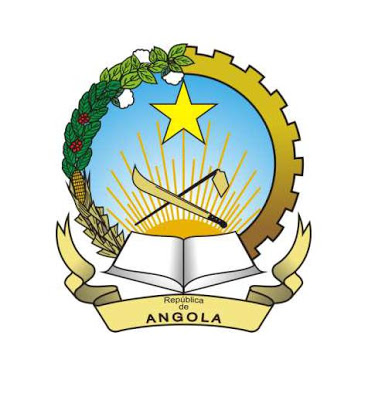
**

**República de Angola**

***Programa de Controlo da Tuberculose***

**PROJECTO:**

**MELHORAR O DIAGNÓSTICO DA DIABETES E DA HIPERTENSÃO NAS PESSOAS COM TUBERCOLOSE**

Data de registo: ………… /…………… /…………………

Unidade de Saúde: Município: ....................................... Distrito: ...........................................

**Dados pessoais**

Nº de Identificação doente: ………………………………………....

Nome completo: ………………………………………………………………………........................... Sexo: F M

Data de nascimento: ……… /……………. / …………….. Idade: ……………………..

Endereço: …………………………………………………………………………………………………………………………………………….

nº ……………. Bairro: ………………………………………. Zona: ……………………

Comuna: ……………………………………………. Município: …………………………………………………………..

Cidade: …………………………………………...... Nº de telefone: ………………………………………...............

Nome do acompanhante: ……………………………………………………………………………………………………………………

Grau de parentesco: ………………………………………. Nº de telefone: …………………………………………………..

**Consentimento do Paciente**

Consente participar neste estudo? Sim Não

Já alguma vez mediste a glicemia? Sim Não

É diabético conhecido? Sim Não

1/4

**Dados sociodemográficos**

Estado civil: Solteiro Casado Divorciado

Viúvo Vive maritalmente

Emprego: Empregado Desempregado Dona de casa

Estudante Outro

Grupos étnicos: Kimbundu Umbundo Bacongo Outro

Religião: Cristã Muçulmana Hindu Outro

Nível de instrução: Analfabeto Instrução primária (1-6 classe)

Instrução secundaria I ciclo ( 7-9 classe )

Instrução secundaria II ciclo ( 10-12 classe)

Universidade

Número de filhos: 0 1 2 ≥3

Rendimento (em Kwanzas por mês): …………………………………………

Número de pessoas que vivem em casa: 1-5 6-8 ≥9

Fuma? Não Sim, ≤ 1 ano Sim, 1-5 anos Sim, ≥10 anos

Usa bebidas alcoólicas? Não Sim, as vezes Sim, todos os dias

**Informação clínica sobre a TB/VIH**

Você já fez tratamentos de TB? Sim Não

Sente um o mais destes sintomas? Tosse Hemoptise Febre

Astenia Dispneia Perda de peso

Suores nocturnos Toracalgia

Tem familiar com TB? Sim Não

Já alguma vez fez o teste de VIH? Sim Não

Está em TARV? Sim Não

Esquema de tratamento………………………………………………………………………………………………………………………

2/4

**Informação clínica sobre diabetes**

Você já ouviu falar da diabetes? Sim Não

Está em tratamento? Sim Não

Tipo de tratamento ………………………………………………………………………………………………………………………………

Há quanto tempo ……………………………………………………………………………………………………………………………….

Tem familiar diabético? Sim Não

Sente um ou mais destes sintomas? Polifagia Poliúria Polidipsia Fraqueza

Já comeu? Sim Não

A que horas? ……………………………………………………………………………………………………………………………………....

**Informação clínica sobre a tensão arterial**

É hipertenso conhecido? Sim Não

Está em tratamento? Sim Não

Tem familiar hipertenso? Sim Não

**Informação clínica adicional**

Tem outras doenças? Sim Não

Qual? …………………………………………………………………………………………………………………………………………………...

……………………………………………………………………………………………………………………………………………………………..

3/4

**Diagnóstico clínico**

Nº de registo de laboratório TB: ……………………………..

Resultado da baciloscopia para TB: BK+ BK− BK N/R

Microscopia: Negativo 1-9 + ++ +++

Resultado do teste para VIH: VIH + VIH – VIH N/R

Tensão arterial: Máximo: ………... mm/Hg Mínimo: ………… mm/hg Pulso: ………….P.

**[V.N. máximo: 140 mm/Hg mínimo: 90 mm/hg]**

Dados antropométricos: Peso: …….......... kg Altura: ….........…. cm

Perímetro abdominal: ............... cm IMC (peso/altura²): ...............

Diabetes: Tipo de teste para diabetes:

Resultado: …….......... mg/dl **[V.N. ˂110 mg/dl]**

**Encaminhamento**

**Se a glicemia é ˃ 126 mg/dl:**

encaminhar o paciente no Centro de Saúde …………………………………………………….....................................

**Se a tensão arterial é ˃ 140/90 mm/Hg**

encaminhar o paciente no Centro de Saúde ..…………………………………………………………………………………….

**Observações**

…………………………………………………………………………………………………………………………………………………………………………………………………………………………………………………………………………………………………………………………

**N.B.: Se o doente não estiver em jejum há 8 horas, executar igualmente o teste e informá-lo que o teste será repetido no dia do levantamento do resultado da baciloscopia e neste dia o doente deve chegar ao Centro em jejum há pelo menos 8 horas.**

**Nome do Responsável** **Assinatura do Responsável**

……………………………………………………………… …………………………………………………………

4/4
